# A Comparative Analysis of Laparoscopic Gastrectomy Versus Laparoscopic-Assisted Gastrectomy: The Overall and Disease-Free Survival

**DOI:** 10.7759/cureus.56730

**Published:** 2024-03-22

**Authors:** Iván Josué Calderón-Canseco, Manuel A Pérez-Turrent, Miguel Ángel Ramírez-García, Sonia Fernández-Ananín, Eduardo María Targarona Soler, María Balagué-Ponz

**Affiliations:** 1 Department of General Surgery, Instituto Mexicano del Seguro Social General Regional Hospital No. 1 "Dr. Carlos Mac Gregor Sanchez Navarro", Mexico City, MEX; 2 Department of General Surgery, Hospital General Dr. Manuel Gea González, Mexico City, MEX; 3 Department of Genetics, National Institute of Neurology and Neurosurgery "Manuel Velasco Suárez", Mexico City, MEX; 4 Gastrointestinal and Hematological Surgical Unit, Hospital de la Santa Creu i Sant Pau, Autonomous University of Barcelona, Barcelona, ESP; 5 Department of Gastrointestinal and Bariatric Surgery, University Hospital Mutua Terrassa, Terrassa, ESP

**Keywords:** disease-free survival, overall survival, laparoscopic-assisted gastrectomy, laparoscopic gastrectomy, gastric cancer

## Abstract

Gastric cancer remains a significant global health challenge with varied survival rates, emphasizing the need for research into effective surgical treatments. In this retrospective study, we compared the 72-month overall and disease-free survival between laparoscopic gastrectomy (LG) and laparoscopic-assisted gastrectomy (AG) in a cohort of 139 patients treated for gastric cancer. The analysis revealed that patients undergoing LG exhibited a significantly higher overall survival rate at 72 months compared to those undergoing AG. Although disease-free survival rates were comparable between the two groups, LG showed a marginal advantage. Subgroup analyses based on the type of gastrectomy and anastomosis demonstrated varied survival probabilities, with laparoscopic-assisted partial gastrectomy yielding the most favorable outcomes. These results highlight the importance of the choice of surgical technique in influencing survival outcomes in gastric cancer.

## Introduction

Gastric cancer ranks as the fifth most common malignancy and the fourth leading cause of cancer-related death worldwide, as reported by the World Health Organization [1]. In our region, it exhibits a prevalence rate of 9.93 per 100,000 individuals [2], with an overall survival rate not exceeding 25% at five years [3].

In Western countries, at least 70% of gastric cancers are diagnosed at locally advanced stages [4,5]. Overall, the prognosis for patients with gastric cancer remains poor, with a five-year survival rate of 20% [6]. In cases of early-detected gastric cancer receiving treatment, the five-year survival rate stands at 90% [7,8]. For more advanced stages following potentially curative surgery, the five-year survival rate ranges between 20% and 30% [9]. In Spain, data compiled by the Spanish Network of Cancer Registries (REDECAN) from 13 population-based cancer registries indicates a five-year net survival rate post-diagnosis of 26.0 for males and 30.3 for females [10].

Surgical resection with curative intent remains the primary therapeutic strategy for gastric cancer [11,12]. Despite advancements in early gastric cancer detection through endoscopy, ultrasound, and computed tomography, a significant number of patients are diagnosed at advanced stages. Due to the aggressive nature and biological characteristics of gastric cancer, infiltration into the serosa or adjacent organs is common, posing challenges in achieving comprehensive resection in cases with locoregional invasion [13]. Post-surgical recurrence is often the leading cause of mortality. Risk factors associated with reduced survival after curative surgical resection of gastric cancer include advanced age, tumor stage, the number of involved lymph nodes, the degree of tumor infiltration into the gastric wall, and the tumor's location [7,14]. Post-surgical recurrence, often distant, is a frequent cause of mortality, although significant rates of locoregional relapses are also reported [15,16].

Perioperative management with chemotherapy for high-risk patients varies globally. In Europe, perioperative chemotherapy is the standard approach; in the United States, chemoradiotherapy is preferred, and in Japan, adjuvant chemotherapy is utilized [6]. This study aimed to compare the 72-month overall and disease-free survival between laparoscopic gastrectomy and laparoscopic-assisted gastrectomy for gastric cancer management.

## Materials and methods

A retrospective analysis was conducted to determine the 72-month overall and disease-free survival in patients diagnosed with gastric cancer treated with laparoscopic gastrectomy at the Hospital de la Santa Creu i Sant Pau. As of 2014, this data pool has been part of the European Registration of Cancer Care (EURECCA) Upper GI Group registry. The study included all cases treated between January 2006 and February 2018. This study was approved by the Institutional Review Board and Ethical Committee for Medical Research of the Health Management Foundation of the Hospital de la Santa Creu i Sant Pau, Barcelona, Spain (#IIBSP-GAS-2018-41).

Patients were categorized into the following two groups based on the type of anastomosis: laparoscopic gastrectomy with intracorporeal anastomosis (LG) and laparoscopic-assisted gastrectomy (AG). Further sub-classification was done based on the type of gastrectomy and anastomosis performed, including total gastrectomy (TG), partial gastrectomy (PG), laparoscopic-assisted total gastrectomy (ATG), and laparoscopic-assisted partial gastrectomy (APG).

Statistical analyses were performed using IBM SPSS Statistics version 25.0 (Armonk, NY: IBM Corp). Categorical variables are presented as frequencies and percentages, while quantitative variables are expressed as means and standard deviations. The log-rank bivariate test and Kaplan-Meier curves were used for overall survival (OS) and recurrence analysis. Additionally, multivariate analysis was conducted using a Cox proportional hazards model, with a significance level set at p≤0.05.

## Results

A total of 139 patients underwent surgery for gastric cancer, with 42.2% (n=59) being female and a mean age of 71.09 years (SD±10.74). Of these, 53.2% (n=74) belonged to the LG group, including 17 who underwent total gastrectomy and 57 partial gastrectomy. In the AG group (n=65), 27 underwent total gastrectomy and 38 partial gastrectomy. Further clinical, epidemiological, and perioperative characteristics, as well as complications, can be found in a previously published article in this journal [17].

**Figure 1:**
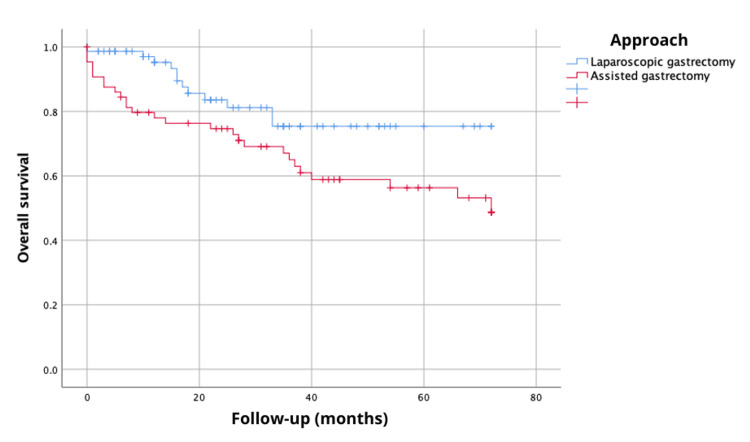
Overall survival at 72 months (six years) according to the type of surgical approach.

**Figure 2:**
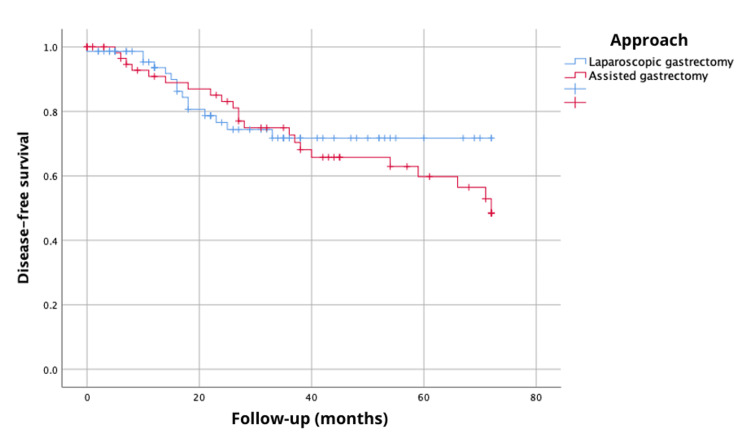
Disease-free survival at 72 months (six years) according to the type of surgical approach.

The analysis of the 72-month overall survival revealed a survival probability of 48.7% with laparoscopic-assisted gastrectomy compared to 75.4% with laparoscopic gastrectomy, showing statistically significant differences via the log-rank test, χ^2^(1)=4.46, p=0.035 (Figure 1). Additionally, disease-free survival at 72 months demonstrated a probability of 48.5% with laparoscopic-assisted gastrectomy and 71.7% with laparoscopic gastrectomy, although this did not reach statistical significance (log-rank, χ^2^{1}=0.25, p=0.61) (Figure 2).

**Figure 3:**
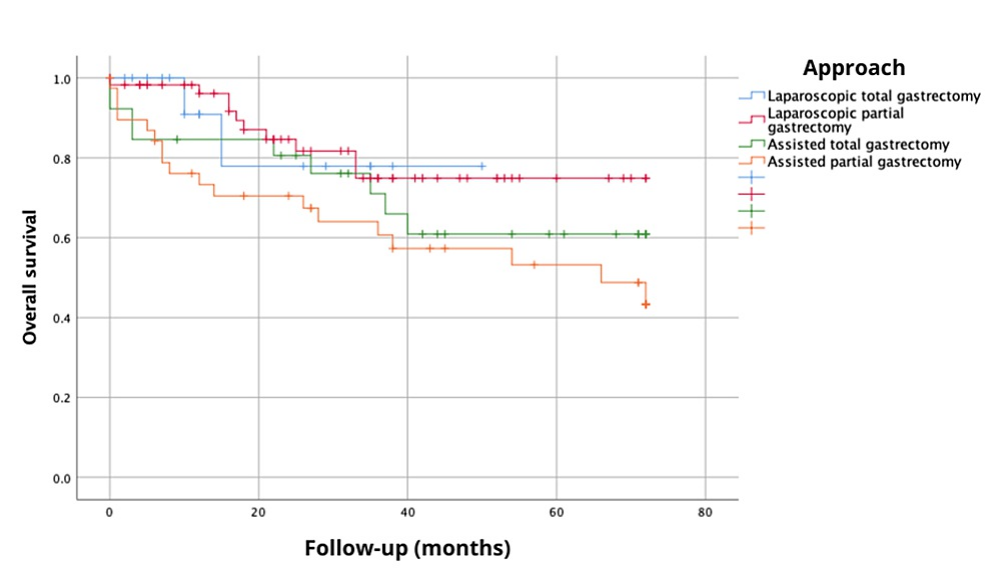
Overall survival at 72 months (six years) considering the type of gastrectomy and anastomosis performed.

**Figure 4:**
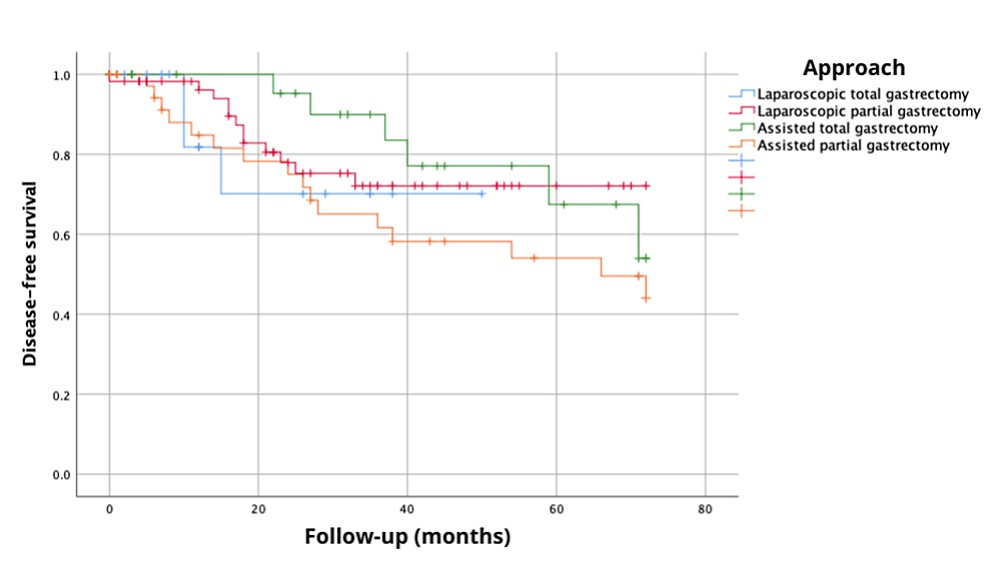
Disease-free survival at 72 months (six years) considering the type of gastrectomy and anastomosis performed.

Furthermore, the analysis of the 72-month overall survival, considering the type of gastrectomy and anastomosis performed (TG, PG, ATG, and APG), showed survival probabilities of 77.9, 74.9, 60.9 and 43.4%, respectively, with no statistically significant differences (log-rank, χ^2^{3}=5.573, p=0.134) (Figure 3). Disease-free survival at 72 months, based on the type of gastrectomy and anastomosis performed (TG, PG, ATG, and APG), exhibited probabilities of 70.1%, 72.1%, 54%, and 44%, respectively, with no statistically significant differences (log-rank, χ^2^{3}=2.72, p=0.436) (Figure 4).

Finally, we utilized a multivariate Cox proportional hazards model to predict mortality in gastric cancer patients, which was found to be statistically significant (χ^2^{6}=34.033, p<0.000). This model identified several key factors influencing mortality risk. Gender was a significant factor, with males having a higher risk compared to females (ExpB 2.16). Age was another critical factor increasing the risk of death 1.046 times for each additional year of age in gastric cancer patients. Recurrence also significantly heightened the risk of death (ExpB 3.521). However, the analysis of different subtypes of anastomosis approaches did not show a statistically significant impact on mortality risk. Other model characteristics (ExpB, 95% CI, and statistical significance) are presented in Table 1.

**Table 1:** Multivariate Cox proportional hazards model for predicting death in gastric cancer. B: coefficient B; SE: standard error; df: degrees of freedom; sig.: statistical significance; CI: confidence intervals

Variables	B	SE	Wald	df	Sig.	ExpB	95.0% CI for expB	
							Inferior	Superior
Sex	0.776	0.391	3.932	1	0.047	2.173	1.009	4.681
Age	0.045	0.019	5.596	1	0.018	1.046	1.008	1.086
Approach	0.440	0.364	1.464	1	0.226	1.552	0.761	3.165
Recurrence	1.256	0.348	13.012	1	0.000	3.510	1.774	6.944

## Discussion

The overall survival of gastric cancer patients has shown improvement over the past two decades. The recent EUROCARE-5 study illustrated a slight increase in five-year survival, from 23.3% between 1999 and 2001 to 25.1% between 2005 and 2007 [18].

Our comprehensive retrospective study, comparing laparoscopic gastrectomy (LG) and laparoscopic-assisted gastrectomy (AG) in gastric cancer patients, has yielded significant insights into the surgical management of this challenging disease. Over a detailed 72-month follow-up period, our findings indicate a superior overall survival rate for patients undergoing LG compared to AG. Specifically, LG patients demonstrated a 71.7% overall survival rate, markedly outperforming the 48.5% observed in AG patients. Moreover, our study revealed comparable disease-free survival rates between the two groups, with a slightly higher tendency in favor of LG.

The disparity in our overall and disease-free survival results, compared to existing literature, raises significant questions [6-9]. Several reasons could account for these discrepancies. Key among these is the unique demographic and clinical profile of our patient cohort, which could influence survival outcomes. Additionally, the specific clinical practices and surgical techniques employed at our institution, which may differ from those reported in other studies, could account for some of these differences. It is also important to consider potential variations in methodology, including patient selection criteria and follow-up protocols, which could contribute to the observed disparities. These factors highlight the importance of contextualizing our results within the broader spectrum of gastric cancer research.

Our study, while providing critical insights into the survival outcomes following various surgical techniques for gastric cancer, is subject to certain limitations that merit consideration. Its retrospective nature, the sample size and scope of the patient cohort, and the differences in clinical practices, surgical techniques, and patient selection criteria within our institution may contribute to outcome variations. Furthermore, the variability in defining clinical events, follow-up procedures, and data collection methods could impact the consistency and reproducibility of our results, while our study contributes important insights into the survival outcomes of gastric cancer post various surgical techniques, these findings should be interpreted with consideration of the aforementioned limitations and the context of existing literature.

## Conclusions

Our investigation into laparoscopic gastrectomy versus laparoscopic-assisted gastrectomy for gastric cancer delineates substantial differences in overall and disease-free survival, it contributes to the evolving landscape of gastric cancer management, emphasizing the potential benefits of laparoscopic techniques in enhancing patient survival. These results suggest that the choice of surgical technique is a crucial factor in improving survival outcomes for gastric cancer patients. However, the variations from existing literature and the inherent limitations of our retrospective study design highlight the need for further research. Future inquiries should scrutinize specific factors influencing survival outcomes, facilitating nuanced treatment strategies. Prospective studies and broader patient cohorts are essential to validate these findings and refine surgical approaches in gastric cancer treatment. 
